# Chinese expert consensus on programming deep brain stimulation for patients with Parkinson’s disease

**DOI:** 10.1186/s40035-018-0116-x

**Published:** 2018-04-30

**Authors:** Shengdi Chen, Guodong Gao, Tao Feng, Jianguo Zhang, The Chinese Medical Association neurosurgery branch of functional neurosurgery group, The Chinese Medical Association neurology branch of Parkinson’s disease, Movement disorders group, The Chinese Physician Association neurosurgeon branch of functional neurosurgery expert committee, The Chinese Physician Association neurologist branch of Parkinson’s disease and Movement disorders professional committee, Chinese expert committee on Deep Brain Stimulation for Parkinson’s Disease, Anmu Xie, Biao Chen, Bomin Sun, Chaoshi Niu, Fangang Meng, Guodong Gao, Haibo Chen, Huifang Shang, Jian Wang, Jianguo Zhang, Jianjun Wu, Jun Wang, Kai Zhang, Ling Chen, Lingjing Jin, Ming Shao, Qin Xiao, Shengdi Chen, Shizhong Zhang, Shujun Xu, Tao Feng, Tao Wang, Wei Wang, Weifeng Luo, Wenbin Zhang, Xiaodong Lu, Xiaowu Hu, Xinhua Wan, Xuelian Wang, Yi Guo, Yiming Liu, Yingqun Tao, Yuqing Zhang, Zhanhua Liang, Zhenfu Wang, Zhipei Ling

**Affiliations:** 1 Department of Neurology, Ruijin Hospital, Shanghai Jiaotong University School of Medicine, Shanghai, 200025 China; 2 Department of Neurosurgery, Tangdu Hospital, Fourth Military Medical University, Xian, 710038 China; 3 Department of Neurology, Beijing Tiantan Hospital, Capital Medical University, Beijing, 100050 China; 4 Department of Neurosurgery, Beijing Tiantan Hospital, Capital Medical University, Beijing, 100050 China

## Abstract

**Background:**

Deep Brain Stimulation (DBS) therapy for the treatment of Parkinson’s Disease (PD) is now a well-established option for some patients. Postoperative standardized programming processes can improve the level of postoperative management and programming, relieve symptoms and improve quality of life.

**Main body:**

In order to improve the quality of the programming, the experts on DBS and PD in neurology and neurosurgery in China reviewed the relevant literatures and combined their own experiences and developed this expert consensus on the programming of deep brain stimulation in patients with PD in China.

**Conclusion:**

This Chinese expert consensus on postoperative programming can standardize and improve postoperative management and programming of DBS for PD.

## Background

Deep Brain Stimulation (DBS) therapy for the treatment of Parkinson’s Disease (PD) is now a well-established option for some patients. An expert consensus in China on DBS in PD was released in 2012 [1, 2], which standardized the indications and processes of DBS therapy, and strengthened the close coordination/cooperation between Neurosurgery and Neurology. In recent years, diagnosis and treatment of PD has made great progress both locally and internationally. The International Parkinson and Movement Disorder Society (MDS) developed a new standard for clinical diagnosis in PD in 2015 [3]. China also developed an diagnostic criteria for PD, which standardized diagnosis and treatment [4] and Chinese PD treatment guidelines (third edition) [5] that regulated the diagnosis and treatment of PD in China.

Postoperative programming is an important part of DBS for PD. In combination with drug therapy, standardized programming processes can identify the best postoperative stimulation parameters, relieve symptoms and improve quality of life. Based on the understanding of the importance of DBS management and programming in PD patients, in order to improve the level of postoperative management and programming, the Chinese Medical Association neurosurgery branch of functional neurosurgery group, the Chinese Medical Association neurology branch of Parkinson’s disease and Movement disorders group, the Chinese Physician Association neurosurgeon branch of functional neurosurgery expert committee and the Chinese Physician Association neurologist branch of Parkinson’s disease and Movement disorders professional committee of national experts, in full review of relevant literature on the basis of the combination of recent years PD diagnosis and DBS therapy of the latest research results, combined with the clinical experience of the expert group to discuss the development of the “*Chinese expert consensus on postoperative programming Deep Brain Stimulation for Parkinson’s disease*”.

## Initial programming

### The time at which the first programming is initiated remains controversial

Most hospitals choose to start programming at 2 to 4 weeks postoperative. However, few other hospitals begin programming during the hospitalization period. Although early programming stimulation can help facilitate treatment of patients, we have to consider the microlesion effect and relatively significant impedance variation following the DBS electrode placement, which usually last weeks or even months postoperatively. Thus, a later programming allows for the microlesion effects to disappear and electrode impedance to become relatively stable. Current-constant stimulation (CCS) can dynamically adjust the voltage to the change of electrode impedance to tissue contact surface, providing more stability in stimulation strength. Although first programming tested under CCS is recommend in some paper [6, 7], whether CCS is better than the traditional voltage-constant stimulation (VCS) is not sure, as the literature directly comparing these two is sparse, with no clear conclusion at least on motor symptoms so far. Before programming, doctors should inform patients that dyskinesia, dizziness, numbness and other stimuli related complications could be experienced during the programming to obtain their understanding and cooperation.

### Initial parameters setting

DBS programming is a difficult and time-consuming process, which is best conducted by a highly trained clinician who understands not only the technical aspects of DBS but also PD-related issues and the management of pharmacological treatments. Importantly, postoperative MRI or CT scans are recommended to identify the electrode location. Prior to the initiation of stimulation, the recording of each electrode’s contact impedance is recommended to troubleshoot hardware problems [8, 9]. These can also be used in future programming as a reference. Generally, the side expressing most severe symptoms is first selected, before proceeding to the contralateral side. Then, one determines for each electrode contact the amplitude threshold for inducing a clinical response and side effects using monopolar stimulation and gradually increase in amplitude (0.2–0.5 V) so as not to cause discomfort. We recommend programmer to participate in or refer to the DBS intraoperative electrophysiology and anatomic target in order to understand the placement of the electrode electrode contacts for postoperative programming.

The initial programming process is as follows: Connect the programmer to the implantable pulse generator. When the connection is complete, input the patient’s basic information and stimulator-related information. To test the impedance of each electrode contact, so as to make sure the connections are intact and then predict the selection of contacts. Test the corresponding contacts one by one to observe the effect on the patient’s symptoms and also note side effects. While monopolar stimulation mode is preferred, bipolar stimulation modes are also considered according to the patient’s response to adjusted stimulation parameters (frequencies, pulse widths, voltage/current). The bipolar setting is often used to limit side effects identified under monopolar stimulation setting where good response is elicited but limited by side effects. An optimal contact is then selected based on the response after a thorough screening programming on each contact, based on the response and side effects profiles.

The first programming is usually performed off medication to prevent any interference on the effects of DBS. The goal is to determine the amplitude threshold for clinical benefits and determine side effects for each of the four electrode contacts [9, 10]. Some centers also exam the patient again after the medication is on during the same visit to make sure that the programmed setting at medication off status does not produce dyskinesia at medication on status.

### Stimulus parameters

There are many published cases and clinical trials that report the most commonly used DBS frequencies, pulse widths and electrode configurations. However, in the clinic there is wide variability that exists between individual patients, meaning they cannot simply receive the given parameters. As a result, the most commonly used parameters should be considered as starting points rather than set end points. Generally, the initial parameters are set to a monopolar mode, with a pulse width of 60 μs and a frequency of 130 Hz, and a stepwise increase in amplitude according to the patient’s response [9–11].

## DBS chronic stimulation

### General principles

Before programming, it is necessary to ask the patient about changes in symptoms since last visit and exam the patients to understand the clinical problem and also get a new baseline to compare with before you re-program them. Also you need check the DBS settings (contact, voltage, pulse width and frequency) and impedance and current before you reprogram them and compare them to the parameters you put on at his/her last visit and make sure that the DBS still works as should be before your reprogramming. Optimization of DBS parameters is usually attained within 3 to 6 months over 4 to 5 programming sessions [8]. The overall goal is to alleviate symptoms and prevent side effects by using minimal stimulation intensity and a minimum dose to obtain the maximal therapeutic efficacy.

### Chronic parameters setting

According to the patient’s condition and response, monopolar stimulation, bipolar stimulation or double monopolar stimulation mode can be chosen. The proportion of monopolar stimulation and double monopolar stimulation settings are increased slightly as time goes on.

The voltage is generally not more than 3.6 V when using original battery, and the voltage of rechargeable stimulator is not subject to this restriction.

The change in voltage for the subthalamic nucleus (STN)-DBS is relatively large but should be less than 3.6 V, while the frequency change should be less than 190 Hz. Using a greater pulse width means that the stimulated nucleus and surrounding structure are affected, which easily induces adverse effects and increases energy consumption. If the voltage is increased to 3.6 V, the narrowest pulse width of 60 μs can be then increased to 90 μs, allowing for a subsequent reduction in voltage to avoid side effects. 60 Hz will be tried in freezing of gait, dysphagia and axial symptoms in patients with freezing of gait at 130 Hz or higher frequency. Generally, if the electrode position is correct, the narrowest pulse width is sufficient. The combination of the highest voltage with the narrowest pulse width is most effective [12]. When patients demonstrate freezing of gait, dysarthria and other midline symptoms, interleaved stimulation, low frequency stimulation(LFS), multiple simulation settings or variable frequency stimulation (VFS) mode are recommended [13–15].

## Important concepts of deep brain stimulation

### VCS and CCS

At present, the Implanted pulse generator (IPG) has two modes: VCS and CCS. VCS refers to the output voltage that is fixed, whereby the current varies with impedance. In contrast, CCS refers to the output current that is fixed, which provides a specific electrical current that automatically adjusts the voltage depending on the impedance. Current studies have shown that CCS is safe for PD patients [16, 17]. Longer follow-up studies are necessary to better clarify the impact of CCS on clinical outcome [18]. VCS is commonly used in China and abroad. However, there is less experience with the use of CCS, meaning uncertainty lies in the relationship between the magnitude of the current value and efficacy of treatment in patients. Generally, brain tissue and electrode interface impedance changes over a short period postoperatively, meaning a CCS is recommended in some paper but still not clear accepted so far [19].

### Interleaved stimulation

With interleaved stimulation, two programs can be interleaved in an alternating fashion on the same lead. Each program drives the same stimulation frequency but different combination of active electrodes, pulse width, and amplitude can be applied. Interleaved stimulation emerges as an effective programming strategy for maximizing symptom control in PD, while regular programming settings do not achieve such favorable results [20]. For different symptoms, two different contacts of interleaved stimulation may have a better effect, but requiring different voltage for the treatment of complex symptoms to find the best balance between the efficacy and adverse reactions [20, 21]. Limited evidence so far, mainly on case report showed that interleaved stimulation may be helpful for gait disorders [22] and dysarthria [23].

### Multiple simulation settings

Physicians can preset multiple simulation settings that can benefit the PD patients on different occasions. Firstly, to meet patient’s needs in different situations. For example, if they feel unsatisfied with their stimulation settings, they could alternate between different settings under the physicians’ instruction. Notably, physicians should always determine the upper limit of the stimulation variable that the patient can use safely and restrict the ability of the patient to exceed that limit. Secondly, the programmer can find the most appropriate stimulation parameters through the “titration” approach. The patient or his caregiver properly operate the patient controller is a prerequisite for using the multiple simulation settings. After a period of time the application program is not effective, the patient can switch to another program group.

### LFS

The DBS stimulation settings that are regularly used are high frequency (> 100 Hz). High frequency stimulation (HFS) improves the cardinal symptoms in patients with Parkinson disease (PD). However, it is less effective at improving the axial symptoms, which include postural instability, gait disorders, speech and swallowing dysfunction. In recent years, LFS has drawn much interest for the improvement of axial symptoms, in comparison with the effects of high frequency stimulation [14, 24], although there are still some studies argued that there was no significant difference between HFS and LFS for controlling symptoms of gait disorders and stability [25]. Thus, some authors still think that further studies are needed for clearly demonstrating the treatment of axial symptoms with LFS in PD patients [26]. Overall, LFS is recommended in patients with freezing of gait at HFS of STN DBS, although LFS could worsen the tremor in small proportion of patients.

### VFS

Tradition stimulation settings drive a constant frequency stimulation, which gives an electrical pulse with constant frequency to the target brain nucleus. High frequency stimulation (HFS) of STN provides long-term improvement of the cardinal signs of PD. However, freezing of gait (FOG) and many other axial symptoms respond poorly to HFS, with symptoms increasing in severity over time. Overall evidence suggests that LFS improves axial symptoms in patients with freezing of gait at HFS of STN DBS, although LFS could worsen the tremor in small proportion of patients. Limited evidence from case report that VFS alternates between low and high frequencies, which may significantly improve freezing of gait and other axial symptoms in PD patients, while maintaining beneficial effects on cardinal symptoms [13]. Further studies, especially large scale randomized controlled clinical trials are needed. The programmer can try to use VFS for gait disorders besides LFS.

### Patient control device

IPG can be adjusted and programmed. Physicians will set the IPG with patient’s basic information and use stimulation settings from the professional programming device. Physicians can allow patients to change certain DBS stimulation variables, such as voltage, and also check the IPG battery or on/off states with their own control device.

### Rechargeable IPG

Commercially available rechargeable IPGs provide a significant advantage in reducing the number of surgeries for IPG replacement. However, there are concerns regarding the safety of using rechargeable IPGs. Patients should strictly follow the instructions. While patients should charge their IPGs in vitro, they should notice the temperature warning to avoid electrical skin burns.

## Common problems and troubleshooting

It must be clear that DBS is an effective supplementary means of PD drug treatment. As a treatment for PD, DBS does not alleviate all symptoms. DBS could significantly improve the cardinal symptoms of PD, including tremor, rigidity and akinesia. However, in the PD patients with axial symptoms, such as postural abnormal gait, balance disorders, dysarthria, dysphagia, the DBS therapy is still challenging. DBS has no therapeutic effect on cognitive impairment and other non-motor symptoms. So taking good care and rehabilitation training may be a better choice. Based on the current level of medical technology development, doctors and patients should have a reasonable expectation on DBS therapy.

### Dyskinesia

Several types of dyskinesia have been identified, which include peak-dose, end-dose and biphasic dyskinesia [27]. Peak-dose dyskinesia manifests after patients take levodopa treatment. End-dose dyskinesia means wearing-off motor fluctuations. Biphasic dyskinesia includes the two types of dyskinesia above.

STN-DBS can induce or aggravate dyskinesia. DBS-induced dyskinesia indicates the accurate location of the implanted electrode and a good prognosis. In most cases, DBS-induced dyskinesia will gradually disappear over several days to several months after turning on the IPG. Physicians can also try different combinations of parameters, choose a bipolar stimulation setting and adjust the dose of levodopa for reducing dyskinesia. Globus pallidus internal (GPi)-DBS has a direct antidyskinetic effect, while STN-DBS depends on the stimulation of dorsal-lateral contact or postoperative reduction of dopaminergic medications [28]. After the control of motor symptoms, PD patients should slowly reduce the dose of levodopa and dopamine receptor agonists to reduce the incidence of dyskinesia. During long-term follow-up, it’s better to maintain stimulation parameters stable, adjustment of anti PD drugs should comply with the “dose titration” principle. If dyskinesia persists, amantadine can be considered [29].

### Dizziness

Dizziness accounts for many reasons and is not limited to symptoms of PD, drug side effects, and side effects of stimulation or other diseases (such as hypotension). It is recommended that there is a reduction in voltage or pulse width, change in stimulation contact, or switch to bipolar stimulation mode (to adjust the electric field range) for alleviating dizziness. If the symptoms still exist after turning off the IPG, the likely cause of dizziness is the adverse drug reactions or effect of other diseases. Therefore, it is recommended to manage drug treatments to reduce dizziness.

### Speech impairment

During disease progression, speech impairment of PD patients laguage will aggravate. It is recommended to attempt to take the following measures to reduce the speech impairments in DBS therapy: 1) reduce the voltage and pulse width of stimulation; 2) use bipolar stimulation; 3) change active electrode contacts; 4) reduce the frequency of stimulation; 5) apply VFS; 6) apply interleaved Stimulation; 7) adjust the dosage of levodopa; 8) undergo speech rehabilitation training. However, these measures have the potential to reduce the effect on the motor symptom, so programmer should weigh the advantages and disadvantages.

### Dysphagia

Dysphagia is an axial symptom, which is inevitable during the natural progression of PD. Dysphagia can induce respiratory problems and is the main cause of death in PD patients. Although STN-DBS is considered to be more likely to induce speech impairments than GPi-DBS, there are no clinical trials that have directly compared the effects of both targets on swallowing function. Due to the progression of PD, it is hard to improve the dysphagia function via adjustment of drugs or DBS programming, which leaves the high quality of nursing care the only choice for symptom control. Nursing care includes eating soft food and methods to assess swallowing function to ensure that there are no breathing problems. Swallowing function can be improved through adjusting the medicine or rehabilitation training. A high quality of nursing care can prevent aspiration of content and induced asphyxia [30]. For these patients with FOG and dysphagia at usual HFS, LFS of 60 Hz could also be tried [14].

### Gait initiation difficulty

Gait disorders caused by FOG respond poorly to HFS. In addition, the severity of gait disorders becomes more prominent as PD progresses. It is found that not all the patients benefit from adjusted medication but could benefit from the LFS. Gait disorders that respond well to levodopa pre-operation usually helps predict the improvement of gait disorders with STN or GPi-DBS surgery. Interleaved stimulation and VFS have been shown to be effective for the improvement of gait disorders [13]. Patients with gait disorders can also benefit from physical therapy and rehabilitation training.

### Balance disorders and gait disorders

Patients with gait disorders need to increase the drug dose during chronic stimulation period. If the dopaminergic drugs are ineffective, doctors may consider amantadine or other drugs [31]. STN-DBS postoperative, doctors should try interleaved stimulation, LFS, VFS or increase the dose of levodopa when patients with FOG [32]. For the patients with balance disorders, the programmer should pay attention to maintaining the bilateral limb muscle tension symmetry as well as DBS parameters and drugs. The dyskinesia, orthostatic hypotension and other factors affecting the balance of patients with other factors should also be considered [33]. The following options can be recommended. 1) reduction of voltage; 2) change in active electrode contacts (i.e. use of a more ventral contact); 3) reduction in frequency; 4) application of VFS; 5) application of interleaved Stimulation.

### Pain

These symptoms should be assessed to ensure pain is caused by PD rather than any other disease the patient may have. There are several subtypes of pain including musculoskeletal pain, dystonia pain, central pain, radicular pain and akathitic pain. These subtypes respond differently to levodopa treatment, DBS surgery, rehabilitation training and other therapeutic methods. PD patients that suffer from pain could benefit from LFS [34]. Some evidence has shown that anti-parkinson drugs relieve musculoskeletal pain, while anti-depressant drugs alleviate central pain. It should also be noted that pain may also be a side effect of anti-parkinson drugs and withdrawal or adjustment of regimens can improve pain relief [35, 36].

### Cognitive impairment

Postoperative the incidence of cognitive impairment is about 0.8–5.1% [29]. The effects of DBS surgery on cognitive function include the effects of surgical microlesiom and DBS stimulation related effects. Due to damage to the basal ganglia-dorsal prefrontal loop, DBS lead trajectory may lead to cognitive decline [37]. Damage to the caudate nucleus in the electrode path can lead to decreased working memory and overall cognitive function decline [38]. Cognitive function impairment was seen in both GPi and STN DBS, but less impaired in GPi than STN [39]. Patient’s speech and semantic fluency may decline after STN-DBS [40]. A systematic cognitive assessment of patients after DBS should include a cognitive assessment in both DBS on and off [29].

For PD patients with cognitive impairment after DBS surgery, the contacts and stimulation parameters should be adjusted [41]. For STN-DBS, contacts should be adjusted to avoid STN non-sensory-motor function subregions [42]. Compared with low-frequency stimulation, HFS leads to a higher risk of language fluency decline [41], so trying to reduce the frequency is a good option. The drugs, including cholinesterase inhibitors (rivastigmine and donepezil) and memantine [43, 44], for cognitive impairment after DBS can refer to the treatment principles of PD cognitive impairment [29, 43]. The caregivers should take good care of PD patients with cognitive impairment [45, 46].

### Postoperative depression, anxiety and apathy

The risk factors for postoperative depression including a history of preoperative depression, rapid or excessive reduction of dopaminergic drugs, and having difficulty to adapt to life changes [47]. Therefore, for DBS patients with postoperative depression, doctors can consider increasing the equivalent dose of levodopa or using dopamine receptor agonist (Pramipexole). When these medical measures prove ineffective, doctors should consider the serotonin reuptake inhibitors and psychotherapy [48, 49]. DBS high frequency stimulation may lead to acute depressive state, which need to be re-programming [47].

Treatment of postoperative anxiety should be based on the principles for PD with anxiety [50]. Benzodiazepine drugs are commonly used anti-anxiety drugs, which may increase cognitive impairment or fall risk. Selective serotonin reuptake inhibitors can also be used in anxiolytic treatment. The incidence of apathy after DBS was related to preoperative non-motor symptom fluctuations, anxiety, young patients and impulse control disorders [51, 52]. Apathy after DBS can be improved by the use of selective D2/D3 receptor agonists [53].

## DBS postoperative oral drug management

The principle of drug application for PD is consistent, regardless of before or after DBS. Doctors should follow the Chinese PD treatment guidelines, refer to the International Movement Disorders Association, the American Academy of Neurology, the European Federation of Neurological Association and other international guidelines and recommended consensus. Meanwhile, the efficacy of DBS and potential adverse reaction risk should also be considered to comprehensively adjust drugs.

DBS postoperative include early postoperative period and long-term follow-up. The DBS perioperative period include the first day to 1 week postoperative. PD patients should take preoperative drugs after anesthesia awareness. It is suggested to maintain the dose of preoperative levedopa in patients with significant preoperative motor symptoms. The dose of levedopa can be reduced appropriately due to the microlesion effect after DBS electrode implantation. The patients with preoperative dyskinesia should reduce the dose of levedopa. Other anti-PD drugs can be suspended to avoid the corresponding adverse reactions. In the early postoperative period (1 week to 4 weeks after operation), the microlesion effect gradually faded, and anti-PD drugs could be gradually adjust to preoperative medication. Levedopa is still recommended to the elderly over the age of 70 for PD patients with preoperative mild cognitive impairment or neuropsychiatric symptoms. For patients with preoperative or postoperative dyskinesia, the equivalent dose of levodopa may be considered to reduce. For PD patients with severe preoperative or postoperative end-of-dose dyskinesia, it’s better to increase the levodopa equivalent daily dose (LEDD) or other anti-PD drugs.

During DBS chronic stimulation period, doctors can determine the type and dose of drugs according to patient’s motor symptoms and non-motor symptoms after the application of conventional DBS parameters. Usually STN-DBS postoperative, LEDD can be reduced by 30–50% [17, 40, 54, 55] (Class 1 Evidence, Class I recommendation). Drugs can be progressively reduced under the guidance of a physician if patient’s symptoms can be alleviated by DBS without severe complications. If the voltage reaches the limit, continued increase will significantly increase energy consumption, except for rechargeable DBS. It should be aware that overly reduced dopaminergic medication postoperatively can cause anxiety and depression. Also increase in voltage could cause side effect, not just because of the energy consumption, regardless if whether rechargeable battery or “not” is added. Due to DBS-related adverse reactions, patients can gradually adjust drugs after reaching a steady state. The selection of drug and dose should refer to patient’s age, occupation and life needs and postoperative symptoms.

## Conclusion

Postoperative management and DBS programming for PD patients are important aspects of DBS therapy, which must be paid full attention. While DBS has been in use for nearly 30 years, there is still no systematic programming guidelines. This has led to inconsistent and inefficient adjustments in stimulation parameters, as well as numerous and unnecessary patient visits. These issues have compelled us to find ways to improve the efficiency of our programming sessions that are aimed at quality improvements, thereby enhancing the patient’s quality of care. Patients should be regularly referred for evaluation and parameters adjustment. We advocate that in the future, establishment of programming database replace the current paper-based programming record for ease of review and sharing experience. In recent years, there are some new progress in DBS programming, such as the directional electrode and advanced DBS software. These new technologies play a huge role in the development of DBS programming, which can be applied under the guidance of doctors. It must be clear that none of the above-mentioned IPGs has all the features to date. Both the doctors, patients and their caregivers should read the DBS equipment instructions in detail to give full play to the role of DBS.

## Abbreviations

CCS: Current-constant stimulation
DBS: Deep brain stimulation
FOG: Freezing of gait
GPi: Globus pallidus internal
HFS: High frequency stimulation
IPG: Implanted pulse generator
LEDD: Levodopa equivalent daily dose
LFS: Low frequency stimulation
MDS: Movement disorder society
PD: Parkinson’s disease
STN: Subthalamic nucleus
VCS: Voltage-constant stimulation
VFS: Variable frequency stimulation
